# Meningitis in a patient with neutropenia due to *Rothia mucilaginosa*: a case report

**DOI:** 10.1186/s13256-018-1947-x

**Published:** 2019-03-12

**Authors:** Maxim Clauwaert, Patrick Druwé, Pieter Depuydt

**Affiliations:** Intensive Care Unit, Universitair Ziekenhuis Gent, C. Heymanslaan 10, 9000 Ghent, Belgium

**Keywords:** *Rothia mucilaginosa*, Meningitis, Acute myeloid leukemia, Neutropenia

## Abstract

**Background:**

*Rothia mucilaginosa* is a Gram-positive bacterium occurring as a commensal in the oral cavity and upper respiratory tract. Although rarely pathogenic in an immunocompetent host, it can cause severe opportunistic infections in immunocompromised individuals.

**Case presentation:**

A 67-year-old white woman had a routine blood analysis before undergoing knee surgery. The results showed leukopenia for which bone marrow examination was performed, showing an underlying acute myeloid leukemia. During the neutropenic phase after a second induction with cytarabine/idarubicin, she developed fever, headaches, and photophobia. Cultures of cerebrospinal fluid were positive for *Rothia mucilaginosa*. Despite full therapy with antibiotics, neurosurgical interventions, and intensive care support, our patient died due to refractory intracranial hypertension and transtentorial herniation.

**Conclusions:**

Meningitis due to *Rothia mucilaginosa* is a rare but potentially lethal infection in patients with neutropenia, and evidence-based guidelines for the treatment of this disease are lacking. We suggest an empirical therapy with amoxicillin/rifampicin until adjustments can be made based on an antibiogram. Intrathecal or intraventricular administration of antibiotics can be considered if neurosurgical access is already obtained because of disease-associated complications.

**Electronic supplementary material:**

The online version of this article (https://doi.org/10.1186/s13256-018-1947-x) contains supplementary material, which is available to authorized users.

## Background

Opportunistic infections are a major cause of morbidity and mortality in patients going through a neutropenic phase following chemotherapy or bone marrow transplantation as a treatment for acute myeloid leukemia (AML) [1]. *Rothia mucilaginosa* is a Gram-positive bacterium that occurs as a commensal in the oral cavity and upper respiratory tract. However, in the context of neutropenia, this bacterium causes invasive infections such as bacteremia, endocarditis, peritonitis, pneumonia, foreign body infections, and meningitis [2]. However, because of its rarity as an invasive pathogen, there is little literature on *Rothia mucilaginosa*. The literature on meningitis caused by this bacterium is limited to a few case descriptions almost exclusively within a pediatric population [1–3]. Here we present an adult patient with neutropenia who developed meningitis with *Rothia mucilaginosa*, showing insufficient response to therapy and further clinical deterioration after admission to our intensive care unit. We hope other clinicians can use this article as a guideline when they are confronted with *Rothia mucilaginosa* meningitis.

## Case presentation

**Fig. 1:**
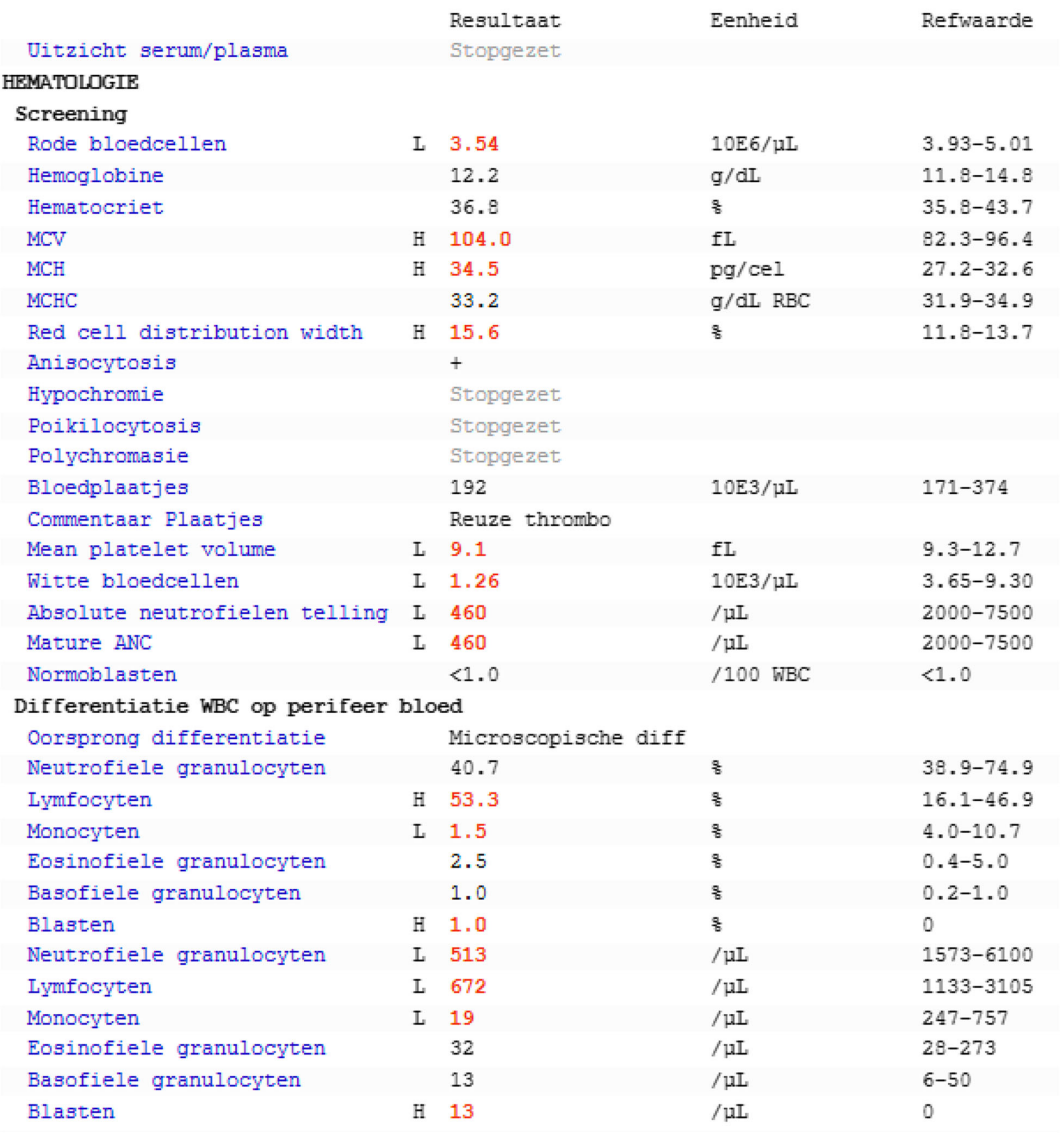
Peripheral blood count at time of diagnosis. ANC: absolute neutrophil count, MCH: mean corpuscular hemoglobin, MCHC: mean corpuscular hemoglobin concentration, MCV: mean corpuscular volume, WBC: white blood cells. Translations: *absolute neutrofielen telling*: absolute neutrophil count, *basofiele granulocyten*: basophils, *blasten*: blasts, *bloedplaatjes*: platelets, *commentaar plaatjes*: comment platelets, *differentiatie*: differentiation, *eenheid*: unit, *eosinofiele granulocyten*: eosinophils, *hematocriet*: hematocrit, *hemoglobine*: hemoglobin, *hypochromie*: hypochromia, *lymfocyten*: lymphocytes, *microscopische*: microscopic, *monocyten*: monocytes, *neutrofiele granulocyten*: neutrophils, *normoblasten*: normoblasts, *perifeer bloed*: peripheral blood, *polychromasie*: polychromasia, *refwaarde*: reference interval, *resultaat*: result, *reuze*: giant, *rode bloedcellen*: red blood cells, *stopgezet*: stopped, *uitzicht serum/plasma*: sight serum/plasma, *witte bloedcellen*: white blood cells

**Fig. 2:**
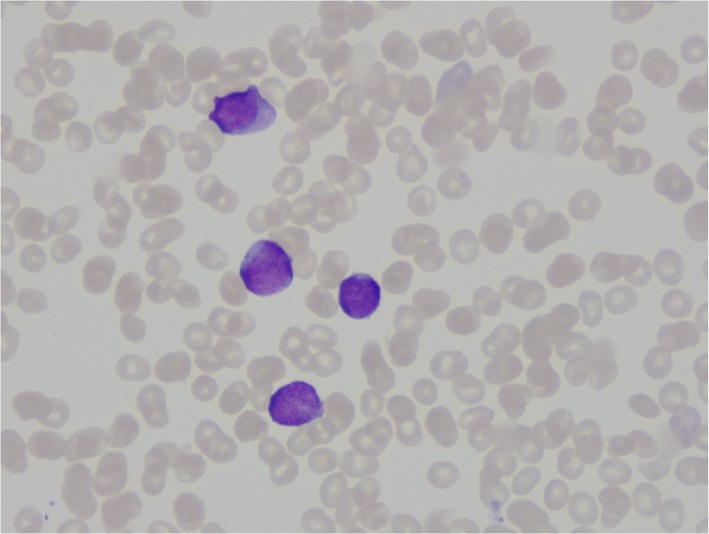
Microscopy of bone marrow aspirate. Because of peripheral blood admixture only peripheral blood cells can be seen. The colored cells are myeloblasts

Our patient was a 67-year-old white woman with no relevant medical history. She was on no medication at time of admission. She did not smoke tobacco and consumed no alcohol. She was retired at time of admission and worked as a librarian before. She was married and had two children, both healthy. Her mother was 88-years old and still alive; her father died at the age of 94, cause not known. None of them had malignancies in their past. At time of admission she felt perfectly normal, had no B-symptoms, no bleedings, and no signs of infections. She was 152 cm in height and weighed 72 kg, with a body surface area of 1.7 m^2^. Her temperature was 36.7 °C (98 °F), blood pressure 128/79 mmHg, and pulse 73 beats per minute. No cardiac, respiratory, gastrointestinal, urological, dental, or neurological anomalies were detected at physical examination. The reason for her admission at our Hematology department was a routine blood analysis, performed before undergoing knee surgery, which showed leukopenia (Fig. 1). A bone marrow examination was performed, revealing an underlying AML (Table 1 and Fig. 2).

**Table 1:** Subtype of leukemia

Cytomorphology	75% blasts, no Auer rods
Flow cytometry (leukemia-associated immunophenotype)	CD15-/CD13-/CD34-/CD117+/CD33+/CD10-/HLADR-/CD45+
Molecular analysis	NPM1+/FLT3-
Genetics	Normal: 46,XX No t(15,17)
Conclusion	AML, favorable risk [9]

*AML* acute myeloid leukemia

**Fig. 3:**
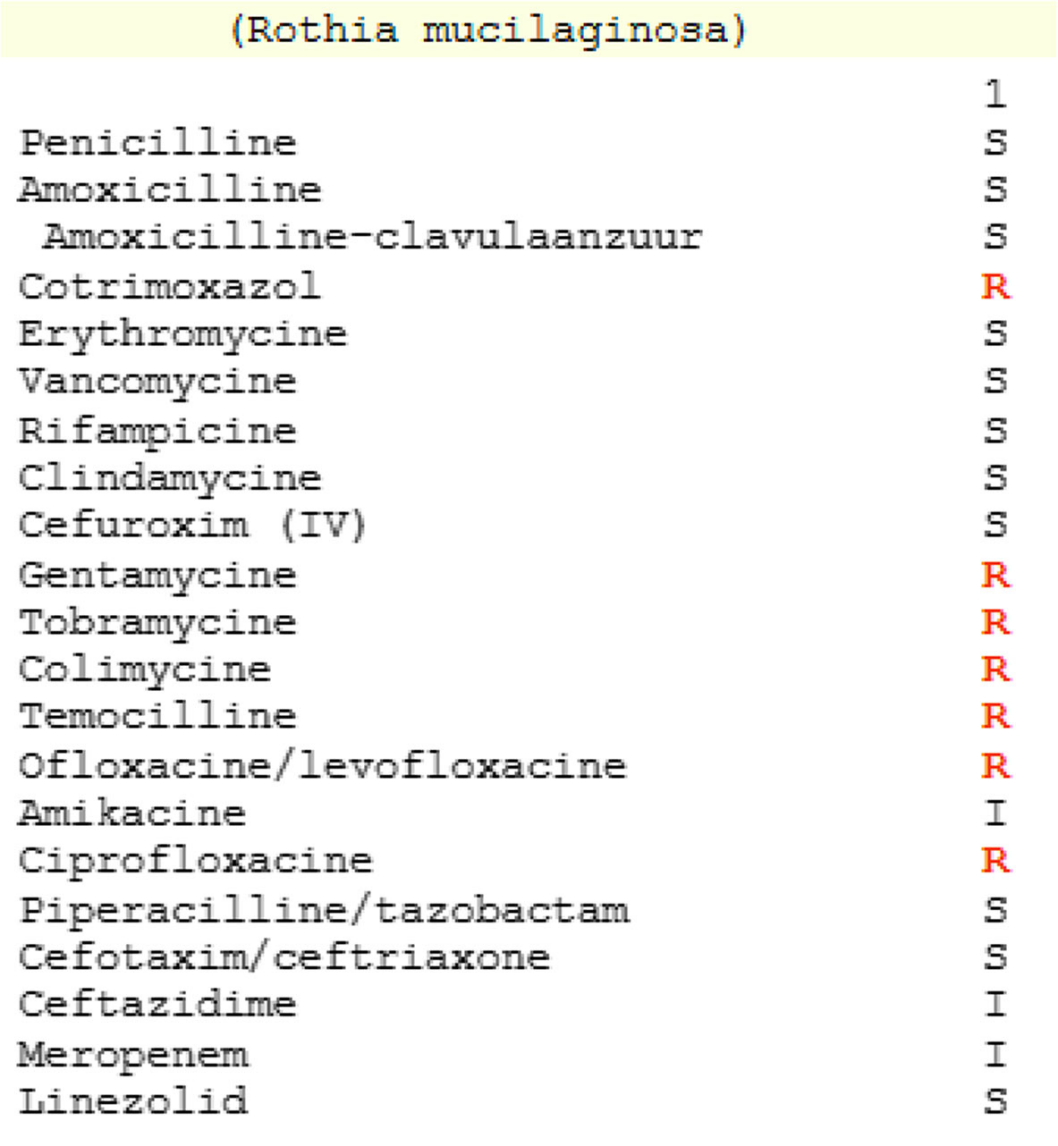
Antibiogram of *Rothia mucilaginosa* at time of diagnosis. *IV* intravenous

**Fig. 4:**
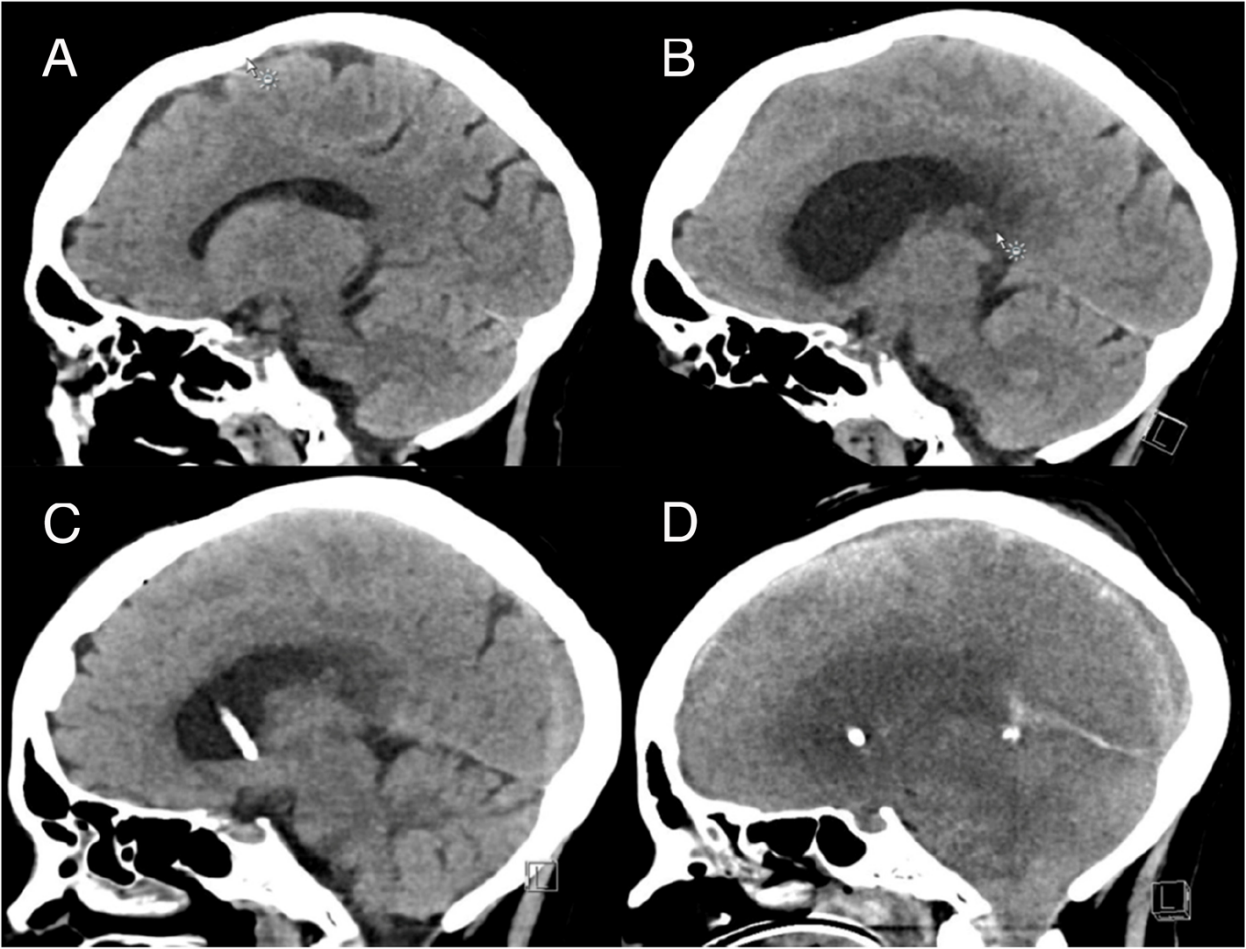
**a** Brain computed tomography at time of diagnosis, without particular complications. **b** Supratentorial hydrocephalus with loss of gyri. **c** Placement of ventriculostomies (bilateral, cannot be seen on the image) with reduction of the volume of the ventricular system. **d** Ventriculostomies *in situ*, although further development of edema and cerebellar herniation

Induction therapy with cytarabine (200 mg/m^2^, continuous infusion over 24 hours, on days 1–7) and idarubicin (12 mg/m^2^, once daily, infusion over 1 hour, on days 1–3) was initiated, and given her neutropenic condition from the start, she stayed in protective isolation at the Hematology department. She received levofloxacin 500 mg once daily for intestinal decontamination and infectious prophylaxis. Since no complications occurred, the second induction was initiated with cytarabine (1000 mg/m^2^, twice daily, infusion over 3 hours, on days 1–3) and idarubicin (12 mg/m^2^, once daily, infusion over 1 hour, on days 3–5). After this therapy, she developed general weakness and diarrhea. At day 35 after the first induction, she developed neutropenic fever for which ceftazidime (2000 mg intravenously every 8 hours, continuous infusion over 8 hours) and Colimycin (colistimethate sodium; 2 × 10^6^ international units intravenously every 6 hours) were started, guided by the presence of a resistant *Pseudomonas aeruginosa* strain in the fecal cultures. Two days later she started vomiting and reported headaches and photophobia. Analysis of her cerebrospinal fluid (CSF) was compatible with bacterial meningitis (Table 2). She was transferred to our intensive care unit receiving empirical antibiotic therapy with ceftazidime, Colimycin (colistimethate sodium), amoxicillin (2000 mg intravenously every 4 hours), and vancomycin (loading dose 1400 mg intravenously over 2 hours, maintenance dose 2500 mg intravenously over 24 hours); although the plasma concentration of this last one only reached therapeutic levels after 2 days. Gram staining on the CSF showed Gram-positive cocci, which were presumed to represent *Staphylococci* at first. However, cultures identified *Rothia mucilaginosa* as the responsible pathogen and after 2 days of empirical therapy, antibiotics were switched to amoxicillin (2000 mg intravenously every 4 hours) and rifampicin (600 mg intravenously every 12 hours) bi-therapy based on an antibiogram (Fig. 3) and the (limited) available literature [1, 2, 4, 5]. Blood cultures remained negative. She was stable for 1 week until she developed significant lethargy and anisocoria. Hydrocephalus was diagnosed with computed tomography (CT) imaging for which external ventricular drainage was provided. Because of a non-communicating hydrocephalus due to ventriculitis, a second drainage was performed. In spite of this, our patient developed bilateral mydriasis without reactivity to light and increasing edema on brain CT, leading to the placement of two more external ventriculostomies in the temporal horns (Fig. 4). Despite these neurosurgical interventions and medical therapy with hypertonic salt and mannitol, intracranial pressure continued to rise, resulting in progressive brain edema and transtentorial herniation. In consultation with her family, further therapy was discontinued and she died on day 49 after the first induction chemotherapy. An autopsy was considered, but, given the clear medical context, this would not have led to other conclusions (Additional file 1).

**Table 2:** Analysis of the cerebrospinal fluid at time of diagnosis

	Result	Reference interval
Glucose (mg/dL)	15.7	49–80
Protein (mg/dL)	77.4	15–50
Lactate (mg/dL)	92.2	10–22
Leukocytes (/μL)	132	< 10
Erythrocytes (/μL)	14.7	0

## Discussion

The fight against aggressive forms of leukemia such as AML, does not only consist of eradicating the malignant cells, but also requires effective treatment of potentially lethal complications of the disease and its antineoplastic therapy. These complications include mainly infectious episodes resulting from the destruction of the immune system [6]. We presented a case of a 67-year-old patient who was diagnosed as having AML and died of an opportunistic infection despite obtaining a molecular remission after the second induction course with chemotherapy. More specifically, she developed meningitis with *Rothia mucilaginosa*, a condition of which little is known and only a few case reports are available and most of them in a pediatric population. We proposed a way to treat this condition because evidence-based guidelines are lacking.

*Rothia mucilaginosa* is a commensal Gram-positive bacterium in the oral cavity and upper respiratory tract. In an immunocompetent host it has low virulence, but it can behave as an opportunistic pathogen in the presence of favorable host factors [2]. Chemotherapy in the treatment of AML not only induces long-term neutropenia, but also extensive mucositis and it requires a central catheter: all these facilitate the invasive character of a germ. In addition, repeated or prolonged exposure to prophylactic or therapeutic broad-spectrum antibiotics may lead to the selection of pathogenic germs [1]. More specifically, a significant association was seen between *Rothia* bacteremia and the use of fluoroquinolones [7]. In our patient, levofloxacin for intestinal decontamination was provided at the start of induction. Although blood cultures remained negative in this case, hematogenous dissemination is assumed.

Diagnosis of meningitis in patients with neutropenia can be delayed because of less pronounced symptomatology as a result of agranulocytosis. In addition, antimicrobial agents penetrate the blood–brain barrier less since there is little or no meningeal inflammation. Because meningitis due to *Rothia mucilaginosa* is rare and only a few cases in an adult population have been described, it was difficult to choose the most adequate therapy. Selection of the antibiotics could be based on the cultures obtained by lumbar puncture. Taking into account the penetration of the agents into the CSF and recommendations from previous case reports, a combination antibiotherapy with amoxicillin and rifampicin was given. Vancomycin had been the first choice in the majority of case reports [1], but a higher minimum inhibitory concentration (MIC) was reported by the laboratory compared to amoxicillin (1 μg/mL versus 0.064 μg/mL). In addition, the penetration of vancomycin in the CSF is thought to be low because of its hydrophilic nature and high molecular weight, although recent research could not confirm this [8]. The question of whether there is additional benefit of intrathecally administered antibiotics is at present unanswered. Given the need for neurosurgical intervention to achieve this, this is, in general, discouraged and only considered when disease complications necessitate a ventricular drainage [1, 4, 5]. However, in our patient, despite the surgical intervention, no intraventricular antibiotics were administered since analysis of the perioperative CSF showed decreasing white blood cell counts as compared to the result at diagnosis, and because of the absence of bacteria on microscopic evaluation. Furthermore, the rapidly declining medical condition of our patient did not allow us to consider any additional intracerebral manipulation.

## Conclusions

*Rothia mucilaginosa* meningitis is a rather rare complication with a high mortality risk in patients with neutropenia. The lack of therapeutic guidelines makes it difficult to maintain evidence-based medicine. Empirical therapy with amoxicillin/rifampicin may be a reasonable initial choice until adjustments can be made based on the antibiogram. Intrathecal or intraventricular administration of antibiotics may be considered if neurosurgical access is already obtained because of disease-associated complications.

## Additional file

Additional file 1: Timeline. (DOCX 57 kb)

## Abbreviations

AML: Acute myeloid leukemia
CSF: Cerebrospinal fluid
CT: Computed tomography
MIC: Minimum inhibitory concentration
