# Supplementary material for: Meningitis in a patient with neutropenia due to Rothia mucilaginosa: a case report
Source: J Med Case Rep. 2019 Mar 12;13:84. doi: 10.1186/s13256-018-1947-x (PMC6413452; doi:10.1186/s13256-018-1947-x)
Supplement: Supplementary file 1 — Timeline. (DOCX 57 kb) [file 13256_2018_1947_MOESM1_ESM.docx]

**Timeline**

| **Date** | **Manifestation** | **Diagnostic testing** | **Intervention** |
| --- | --- | --- | --- |
| 18 sep. 2017 | AML |  |  |
| 19 sep. 2017 |  |  | Induction 1 (cytarabine / idarubicin) |
| 11 oct. 2017 |  |  | Induction 2 (cytarabine / idarubicin) |
| 23 oct. 2017 | Neutropenic fever |  | ceftazidime/colimycin |
| 25 oct. 2017 | Headaches and photophobia | Lumbar punction: diagnosis of bacterial menigitis | - Transfer to ICU  - ceftazidime / colimycin / amoxicillin / vancomycin |
| 27 oct. 2017 |  | Cultures of CSF: *Rothia mucilaginosa* | switch to amoxicillin / rifampicin |
| 3 nov. 2017 | Lethargy and anisocoria | CT: hydrocephalus | External ventricular drainage |
| 4 nov. 2017 | Clinically deteriorating | CT: non-communicating hydrocephalus (ventriculitis) | Second ventricular drainage |
| 5 nov. 2017 | Bilateral mydriasis | CT: increasing oedema | Two more external ventriculostomies |
| 6 nov. 2017 | No results of therapy | CT: transtentorial herniation | Stop therapy |
| 7 nov. 2017 | Patient died |  |  |
